# Improvement of the cell viability of hepatocytes cultured in three-dimensional collagen gels using pump-free perfusion driven by water level difference

**DOI:** 10.1038/s41598-022-24423-y

**Published:** 2022-11-24

**Authors:** Sumire Ishida-Ishihara, Ryota Takada, Kazuya Furusawa, Seiichiro Ishihara, Hisashi Haga

**Affiliations:** 1 Department of Functional Life Sciences, Faculty of Advanced Life Science, Hokkaido University, N21-W11, Kita-Ku, Sapporo, 001-0021 Japan; 2 Division of Life Science, Graduate School of Life Science, Hokkaido University, N10-W8, Kita-Ku, Sapporo, 060-0810 Japan; 3 Faculty of Environmental and Information Sciences, Fukui University of Technology, Gakuen 3-6-1, Fukui, 910-8505 Japan; 4 Department of Advanced Transdisciplinary Sciences, Faculty of Advanced Life Science, Hokkaido University, N10-W8, Kita-Ku, Sapporo, 060-0810 Japan; 5 Soft Matter GI-CoRE, Hokkaido University, N21-W11, Kita-Ku, Sapporo, 001-0021 Japan; 6 Hokkaido University, Room 2-612, Science Bld., N10-W8, Kita-Ku, Sapporo, 060-0810 Japan; 7 Hokkaido University, Room 2-602, Science Bld., N10-W8, Kita-Ku, Sapporo, 060-0810 Japan

**Keywords:** Cell biology, Biomimetics

## Abstract

Cell-containing collagen gels are one of the materials employed in tissue engineering and drug testing. A collagen gel is a useful three-dimensional (3D) scaffold that improves various cell functions compared to traditional two-dimensional plastic substrates. However, owing to poor nutrient availability, cells are not viable in thick collagen gels. Perfusion is an effective method for supplying nutrients to the gel. In this study, we maintained hepatocytes embedded in a 3D collagen gel using a simple pump-free perfusion cell culture system with ordinary cell culture products. Flow was generated by the difference in water level in the culture medium. Hepatocytes were found to be viable in a collagen gel of thickness 3.26 (± 0.16 S.E.)-mm for 3 days. In addition, hepatocytes had improved proliferation and gene expression related to liver function in a 3D collagen gel compared to a 2D culture dish. These findings indicate that our perfusion method is useful for investigating the cellular functions of 3D hydrogels.

## Introduction

The formation of cell-containing biomaterials is one of the approaches used in tissue engineering for the preparation of bioequivalent organs and is of remarkable interest for applications in cell metabolism testing and drug cytotoxicity^1–3^. Three-dimensional (3D) materials, such as decellularized tissues^4,5^ and hydrogels^6^ are used as scaffolds. Hydrogels, which are cross-linked polymer networks containing large amounts of water, exhibit distinct usefulness as 3D scaffolds for cell culture. In particular, collagen hydrogels possess high biocompatibility and can act as implantable scaffolds for bioengineering^6,7^. Indeed, collagen-based bioengineering scaffolds were developed in many previous studies^8–10^. Collagen gels not only function as scaffolds, but also modulate cell functions. 3D collagen gels, which surround cells, can maintain cell viability, proliferation, and physiological activity compared to traditional two-dimensional (2D) substrates, in which cells are seeded on materials, such as tissue culture plastic dishes^11–14^. Cells cultured on a 2D substrate show flat and elongated morphology because they can only expand as a monolayer; on the other hand, cells cultured in a 3D matrix show natural shapes^15^. 3D culture also suppresses the unusually rapid proliferation seen on 2D substrates so it better reflects in vivo proliferation rates^15^. In addition, differentiation of the embryonic body to hepatocytes is accelerated when embedded into 3D collagen gels compared to when seeded onto collagen-coated dishes^16^. Hepatocyte-specific genes were expressed earlier and were more highly expressed in 3D collagen gel cultures, compared to 2D cultures. Furthermore, it has been shown that hepatocyte-like architecture, including developed Golgi apparatus and intercellular canaliculi, is observed in embryonic stem cells cultured in 3D collagen gels^16^.

Although 3D collagen gels have many advantages, one of the key challenges of their use for cell culture is the preservation of viable cells inside the gels. The diffusion of oxygen and nutrients in a hydrogel is limited by distances of 100–200 μm^17,18^; therefore, cell death often occurs in deeper regions of thick gel scaffolds^11^. Perfusion, which is defined as the continuous flow of the culture medium through the hydrogel, is one of the traditional approaches to overcome diffusion limitation^19,20^. However, perfusion systems are not typically used in biological laboratories. Generally, bioengineering researchers develop their own perfusion systems with pumps, special chambers, and various equipment to control the flow rate and adjust mass transport, temperature, and oxygen for cells in 3D scaffolds^21–23^. As a result, the attempt of perfusion cell cultures becomes challenging for biological researchers. Although various perfusion devices are commercially available, skillfully handling highly technical devices is a difficult task for researchers, ultimately serving as a big hurdle to the first attempt at perfusion culture in a common biological laboratory^24^. In some previous studies, Transwell and water level differences were employed to perfuse cell culture medium through a 3D matrix^25–28^. These studies either revealed the crawling out of cells from the gel or compared cell migration in the gel; however, these studies did not evaluate the viability of cells in a thick matrix. We opted to employ perfusion culture to improve cell viability in a thick collagen gel.

Isolated and cultured hepatocytes in vitro are important tools for studies on the liver^29^. HepG2 cells are derived from human hepatocarcinoma and are commonly used for in vitro liver study^30^. HepG2 cells are non-tumorigenic cells that exhibit an epithelial-like morphology and perform many differentiated hepatic functions, such as the production of albumin^31^. Metabolism is an important indicator of liver function. Hepatocytes metabolize various compounds, including drugs. Cytochrome P450 (CYP) enzymes play major roles in drug metabolisms^32^. The functions of hepatocytes are known to be upregulated in cells embedded in a 3D collagen gel compared to those maintained on 2D scaffolds^33,34^.

In the present study, the viability of hepatocytes cultured in 3D hydrogels was improved using a pump-free perfusion cell culture method with ordinary cell culture products. Flow was generated by the difference in water level in the culture medium, and persisted for 12 or 24 h per medium change. HepG2 cells were found to be viable at the central part of the collagen gel of thickness 3.26 (± 0.16 S.E.)-mm in the perfusion culture for 3 days. In addition, hepatocytes had improved proliferation and expression of genes related to liver function in a 3D collagen gel compared to those in a 2D plastic culture dish. These results demonstrate the usefulness of this perfusion method for investigating the functions of cultured cells in a 3D hydrogel.

## Results

### Improved cell viability by medium perfusion using difference in water level

**Figure 1:**
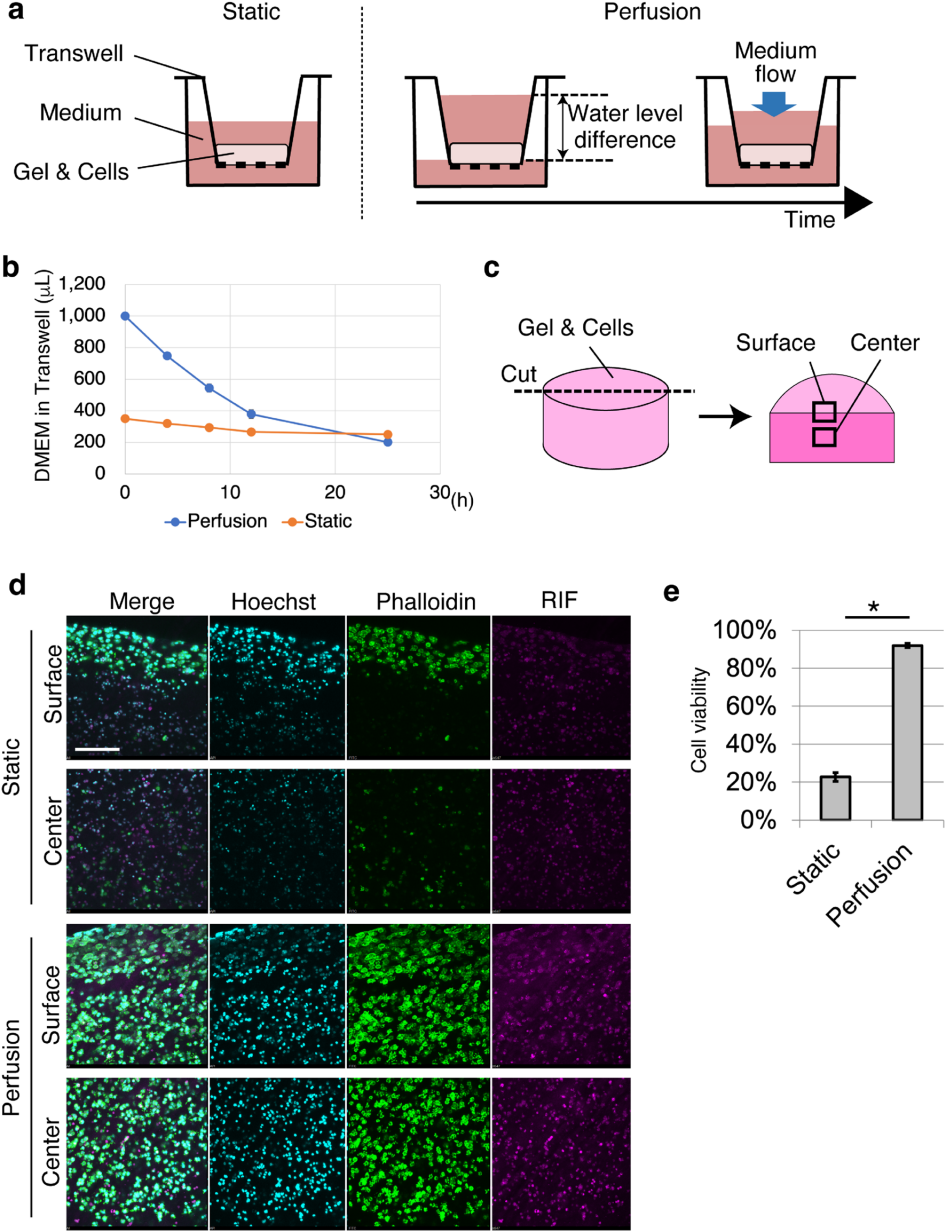
Cell viability improved with perfusion cell culture by water level difference (PCC-WLD). (**a**) Schematic of perfusion cell culture by water level difference (PCC-WLD) and control (static). (**b**) Flow rate of DMEM through the Transwell. *n* = 3 independent experiments. Mean ± S.E. (**c**) Schematic image of sample cutting. Dot line indicates where the samples were cut. Surface and central part of the cross section were observed. (**d**) Fluorescent images of the static and perfusion cell culture based on the live/dead cell assay. Cyan, Hoechst. Green, phalloidin Magenta, reflection interference (RIF). Scale bar, 200 μm. (**e**) The analysis of (**d**). *n* = at least 8 fields from 3 independent experiments. Mean ± S.E. *p < 0.0001 by Student’s *t* test.

**Figure 2:**
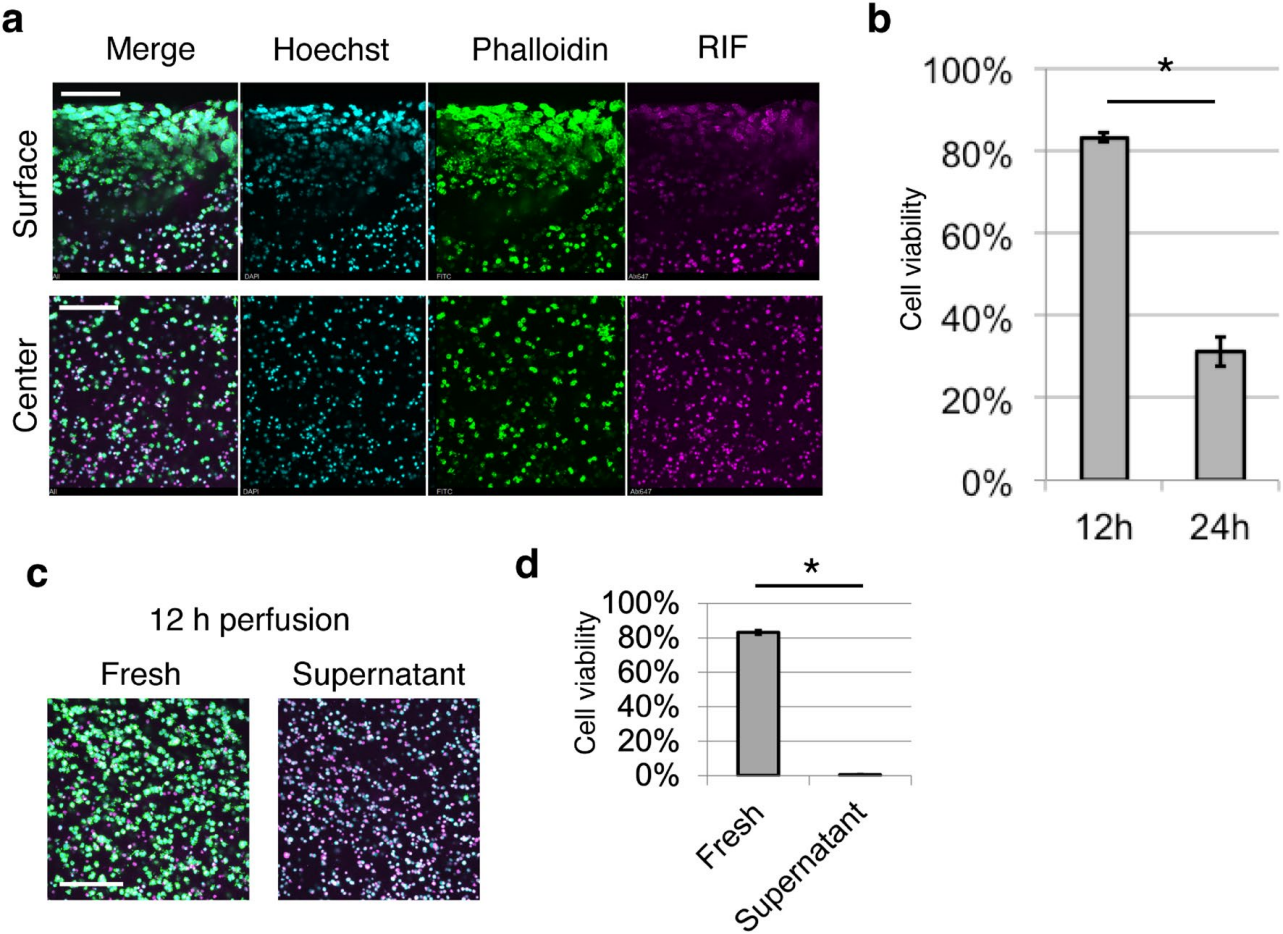
Continuous flow of fresh medium is important for the maintenance of cell viability. (**a**) Fluorescent images after 3 days of culture with PCC-WLD based on the live/dead assay. The medium was replaced every 24 h. Scale bar, 200 μm. (**b**) Analysis of the live/dead assay between 12 and 24 h medium replacement. (**c,d**) Fluorescent images (**c**) and analysis (**d**) of the perfusion culture with fresh or supernatant medium based on live/dead cell assay. The water level difference was restored every 12 h for 3 days. (**a,c**) Cyan, Hoechst. Green, phalloidin. Magenta, RIF. Scale bar, 200 μm. (**b,d**) *n* = 9 fields from 3 independent experiments. Mean ± S.E. *p < 0.0001 by Welch’s *t* test.

A perfusion cell culture system without pumps was established using a cell-containing collagen gel^25–28^. Thereafter, the viable cells in the gel were observed. Briefly, 200 μL of the mixture of HepG2 and collagen gel was placed in a 24-well Transwell (Fig. 1a). The thickness of the cell containing collagen gel was 3.26 (± 0.16 S.E.) mm. To perfuse the culture medium, the interior of the Transwell was filled with medium to create a difference in the water level. For the control, the water level was set to be equal between the interior and exterior of the Transwell. In the presence of a difference in water level, medium in the interior of the Transwell constantly flowed through a permeable membrane at 54.0 (± 1.6 S.E.) μL/h for 12 h (Fig. 1b). To enable continuous flow of the medium, medium was replenished every 12 h for 3 days, and the cell viability was investigated. To observe cell viability in deeper regions from the gel surface, the gel was cut to expose the central part of the collagen gel (Fig. 1c) owing to the limited focal depth of the microscope. Of note, cutting the gel will kill living cells. Further, as the collagen gel was very soft, easy deformation occurred when the gel was removed from the Transwell without fixation. Therefore, the samples were fixed before cutting. To distinguish between live and dead cells after fixation, F-actin, which is uniformly expressed in live cells, was detected. To determine whether F-actin staining can distinguish between live and dead cells, the cells were embedded in a 125 μm-thick gel (250 μL of cell-gel mixture in a glass bottom dish with a diameter of 1.6 mm) and then irradiated with ultraviolet rays (UV). The cells were stained with the dead cell staining dye, SYTOX Green, without fixation. Almost all UV-irradiated cells were SYTOX Green-positive and almost all non-treated (NT) cells were SYTOX Green negative (Fig. S1), confirming the death of the UV-irradiated cells. Subsequently, the UV-irradiated cells and NT cells were fixed and then stained with the F-actin-staining dye, phalloidin. Most UV-irradiated cells were negative for F-actin, and almost all NT cells were positive for phalloidin. Based on these results, phalloidin can be used as a live-cell marker. Both live and dead cells were visible using Hoechst staining and/or reflection interference (RIF) observation. Therefore, the total cells in the observed area were counted using the Hoechst and RIF images. Viable cells were observed in the perfusion system using this staining strategy. After 3 days of culture under static conditions, almost all cells at the surface were alive, while only 22.7 (± 0.02 S.E.)% of the cells in the central part were alive (Fig. 1d,e). However, when using perfusion cell culture with a water level difference (PCC-WLD), cell viability at the center of the gel significantly increased to 92.0 ± 0.01 S.E.)%. To determine the importance of nutrient supply for cell viability, the frequency of the medium change was decreased from every 12 h to every 24 h, which led to a 12 h-perfusing and 12 h-static condition (Fig. 1b). After 3 days of culture, media replacement every 24 h significantly reduced cell viability (Fig. 2a,b). In addition, the supernatant was continuously perfused and media were collected from the dish-cultured HepG2 cells for 24 h to observe their viability. The viability of the supernatant medium was found to be significantly lower than that of the fresh medium (Fig. 2c,d). These results indicate that the supply of nutrients and/or removal of waste products is critical for retaining cell viability within a thick collagen gel. Our findings also indicate that the PCC-WLD improved cell viability in a thick collagen gel by supplying fresh medium.

In addition to HepG2 cells, we examined other cell lines often used in tissue engineering. Mesenchymal stem cells (MSCs) are proliferative and have the potential to differentiate into several cell types, including adipocytes and osteocytes^35^. Human dermal fibroblasts (HDFs), which have recently been reported to show the same properties as MSCs, such as cell surface markers and differentiation potential^35^, were embedded in a collagen gel and cultured under static conditions. Although the cell density was one-tenth that of HepG2 cells cultured in the Transwell, the HDF-containing collagen gel shrank and detached from the cell culture vessel (Fig. S2a). Because the cell-free gel did not shrink, gel shrinkage may have been caused by the contractile force of HDFs^36^. Gel shrinkage causes an interspace between the Transwell and the gel. In this case, the medium was thought to flow through the interspace and not through the gel. Thus, a perfusion system in a Transwell setup may not be appropriate for HDFs. To investigate the viability of HDFs in thick collagen gels, instead of the PCC-WLD system, we embedded the cells into a 3 mm thick collagen gel and incubated the gel in floating culture while shaking. In the control sample of HepG2 cells, the gels did not shrink dramatically, and cells were dead at the center of the gel, indicating that the nutrition supply was insufficient (Fig. S2b–d). On the other hand, for HDFs the gel shrank drastically. Fluorescent images of the cell viability assay showed that the cell density was much higher in HDF cultures than that of HepG2 cells owing to gel shrinkage, and HDFs were alive at the center of the gel. These results indicate that HDFs survive in thick collagen gels without continuous perfusion. Whether the PCC-WLD system applies to other stem cells will be investigated in future work.

### PCC-WLD using a syringe for continuous medium perfusion with fewer medium replacement

**Figure 3:**
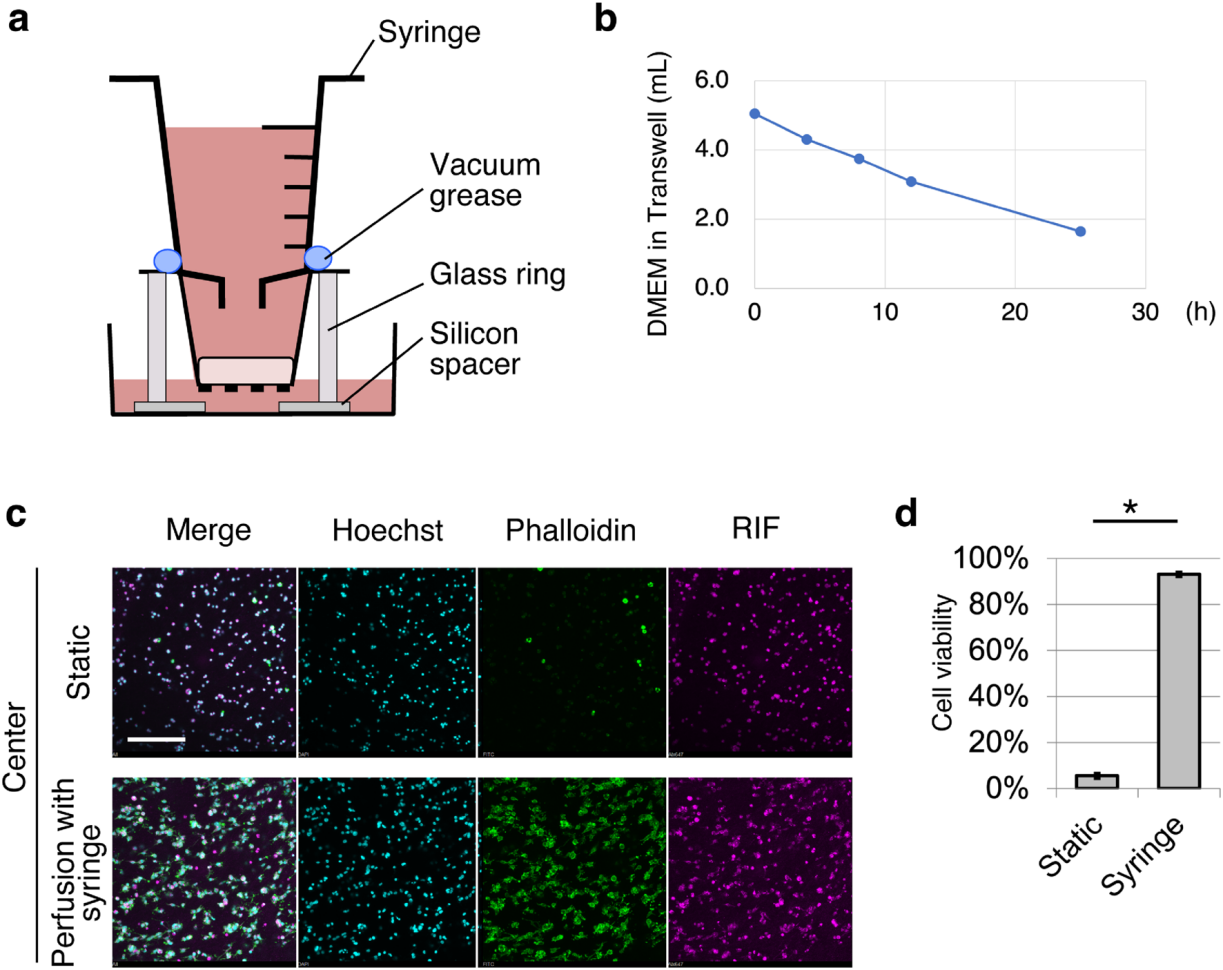
Cell viability improved with PCC-WLD with a syringe. (**a**) Schematic of PCC-WLD with a syringe. (**b**) Flow rate of DMEM from the Transwell in PCC-WLD with a syringe. *n* = 3 independent experiments. (**c,d**) Fluorescent images (**c**) and analysis (**d**) of the live/dead cell assay. Cells were cultured for 3 days. *n* = 9 fields from 3 independent experiments. Cyan, Hoechst. Green, phalloidin. Magenta, RIF. Scale bar, 200 μm. Mean ± S.E., *p < 0.0001 by Student’s *t* test.

**Figure 4:**
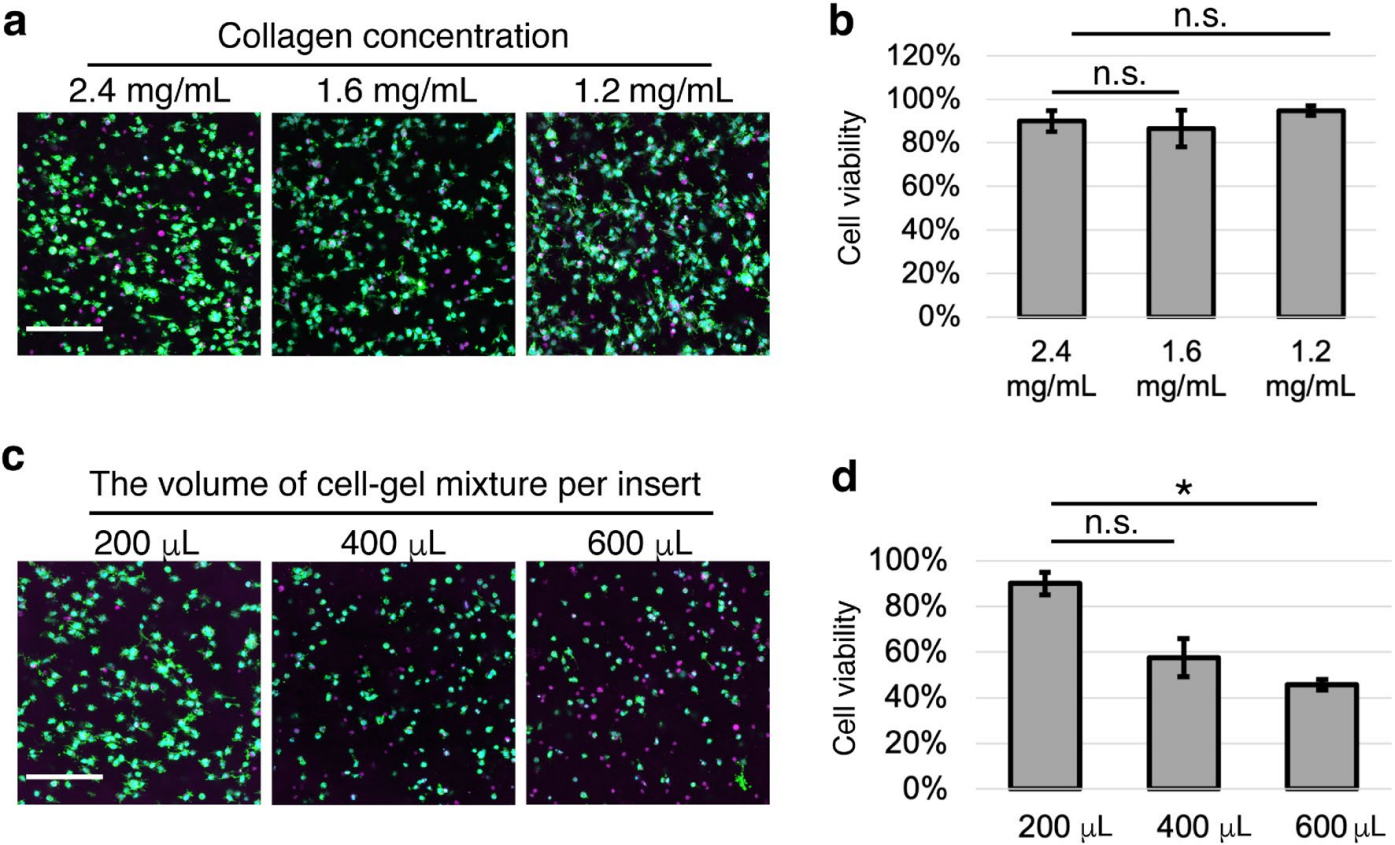
Gel volume, but not collagen concentration, affected cell viability. (**a, b**) Fluorescent images (**a**) and analysis (**b**) of cells embedded in 200 μL of gel with different concentrations of collagen based on the live/dead cell assay. (**c,d**) Fluorescent images (**c**) and analysis (**d**) of cells embedded in 2.4 mg/mL collagen gels with different volumes based on the live/dead cell assay. Cells were cultured for 3 days with PCC-WLD with a syringe. The center of the gel was observed. Cyan, Hoechst. Green, phalloidin. Magenta, RIF. Scale bar, 200 μm. *n* = 3 fields from 3 independent experiments. Mean ± S.E. *n.s.* no significance. *p < 0.05 by Student’s *t*-test with Bonferroni correction.

To maintain a continuous flow in the PCC-WLD, the medium was changed every 12 h. To reduce the frequency of medium change, we improved the PCC-WLD to maintain a continuous flow by changing the medium every 24 h. The 5 mL-plastic syringe was connected to a 24-well Transwell with vacuum grease to increase the volume of the culture medium of the Transwell (Fig. 3a, Fig. S3). As the volume of the medium increased, the 24-well Transwell was placed in a 6-well plate to avoid overflow of the waste medium. A hand-made glass ring with a diameter of 24-wells was used to support the syringe-attached Transwell. The silicon spacer was partially placed under the glass ring to create a space for the waterway. To avoid contamination, the outside of the glass ring was wrapped with UV-irradiated aluminum foil (Fig. S3). By using this system, medium continuously flowed at a rate of 143.7 (± 3.5 S.E.) μL/h over 24 h (Fig. 3b). In addition, after 3 days of culture, cell viability significantly increased compared to that in the static cell culture (Fig. 3c,d). To explore the available experimental conditions for PCC-WLD, we examined the viability of cells in collagen gels using different collagen concentrations, gel thicknesses, and culture periods. Collagen gels were prepared using 200 μL of 2.4 mg/mL collagen when specific instructions were not provided. The decrement of collagen concentration to 1.6 and 1.2 mg/mL did not affect the cell viability (Fig. 4a,b). However, increasing the gel thickness by increasing the gel volume decreased the viability in the central region (Fig. 4c,d) (Fig. S4a). When the gel was observed at approximately equal intervals from the surface, cell viability in the 400- and 600-μL gels decreased according to the depth from the surface of the gel compared to that in the 200-μL gel (Fig. S4b). Thus, the upper limit of the gel volume may be between 200 and 400 μL in our PCC-WLD with a syringe system. We also attempted 7 days of culture with 200 μL of the 2.4 mg/mL gel. The flow rate of the cell-embedded gel decreased significantly on day 7 (Fig. S4c), and the gel-containing cells became thin compared with the cell-free gel (Fig. S4d). Cells in the thin gel were tightly packed. Phalloidin staining revealed live cells around the gel surface; however, cells in the central part of the gel were dead in the PCC-WLD system. These results imply that cells cause compaction of the gel, and compaction causes cell death at the center of the gel. These results indicate that PCC-WLD with syringe improved cell viability and reduced the frequency of medium change; however, more improvements are needed to enable the use of thicker gels and long-term culture.

### Improvement of HepG2 cell proliferation and liver function using a 3D culture with PCC-WLD

**Figure 5:**
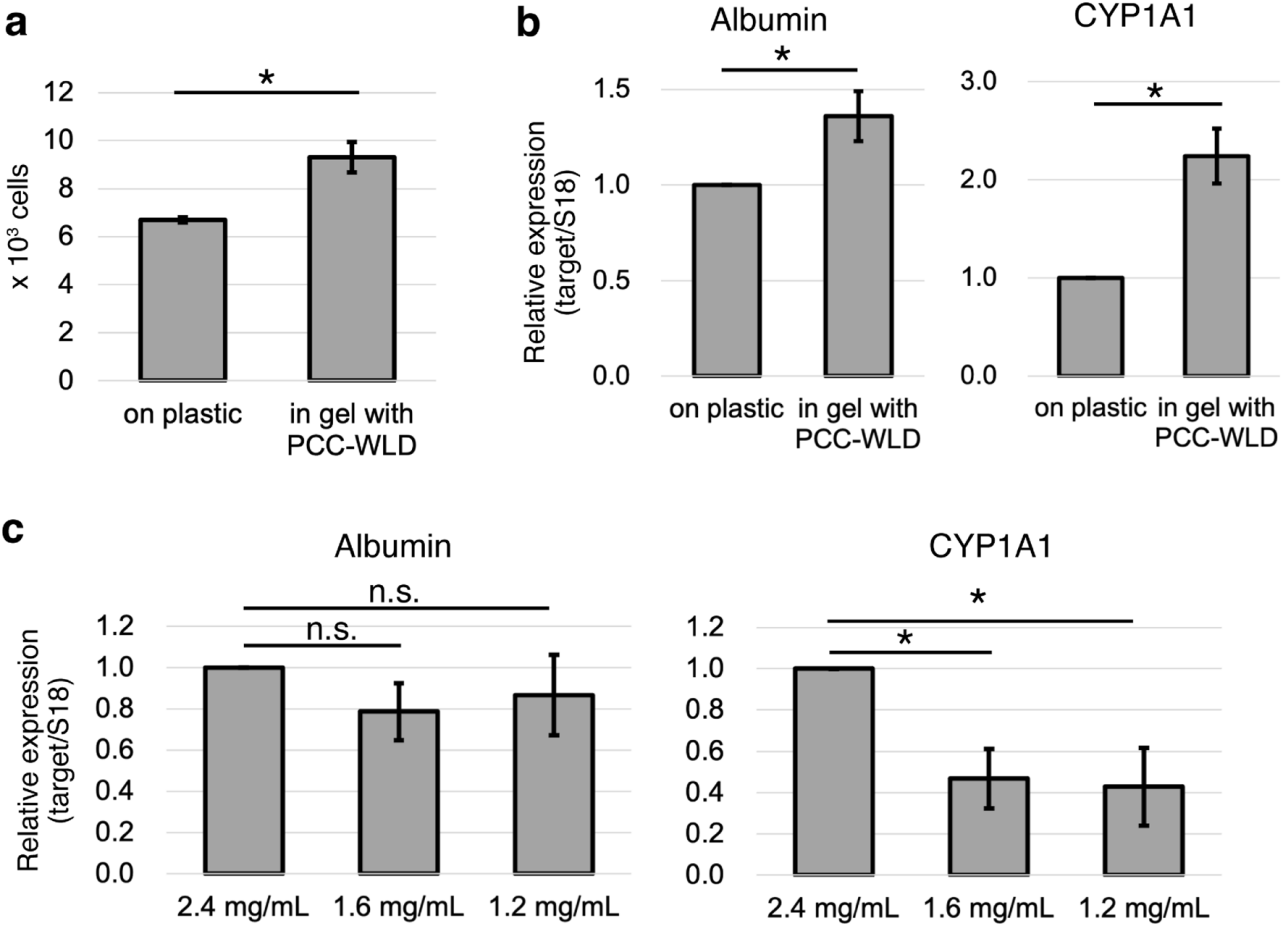
Cell proliferation and gene expression related to liver function were improved with PCC-WLD. (**a**,**b**) Cells were cultured on a plastic dish (static condition) or in 200 μL of 2.4 mg/ml collagen gel (PCC-WLD with syringe) for 3 days. (**a**) Proliferation assay. *n* = 3 independent experiments. Mean ± S.E. *p < 0.05 by Student’s *t* test. (**b**) Quantitative real-time PCR of albumin and CYP1A1. *n* = 3 independent experiments. Mean ± S.E. *p < 0.01 based on the confidence interval (CI) test. (**c**) Quantitative real-time PCR of albumin and CYP1A1 in the cells cultured in 200 μL of gels with different concentrations of collagen. *n* = at least 4 independent experiments Mean ± S.E. *n.s.* no significance. *p < 0.01 based on the CI test with Bonferroni correction.

We proceeded to determine whether the proliferation and liver function of HepG2 cells embedded in a collagen gel with perfusion were improved compared to those of cells on a collagen-coated plastic dish under static conditions. First, the proliferation of HepG2 cells was compared between the cells in a gel with PCC-WLD and those on a plastic dish under static conditions. After 3 days of culture, the cell number was counted following Hoechst staining. The proliferation rate of HepG2 cells in the gel increased by 1.39-fold relative to that on plastic (Fig. 5a). Thereafter, the mRNA levels of albumin and CYP1A1 were determined. Albumin is the most abundant serum protein produced by hepatocytes^37^. CYP1A1 is an important enzyme involved in drug metabolism in the liver^32^. The transcriptional levels of both albumin and CYP1A1 were upregulated in HepG2 cells in 3D culture with PCC-WLD compared to those in a 2D plastic dish (Fig. 5b). Interestingly, the decrease in collagen concentration in 3D culture with PCC-WLD did not affect albumin mRNA expression, but decreased CYP1A1 mRNA expression (Fig. 5c). These results indicate that 3D culture with the PCC-WLD system not only keeps the cells alive in an approximately 3-mm thick collagen gel, but also induces better proliferation and liver function of HepG2 cells than those of cells on a traditional plastic culture dish.

## Discussion

In this study, the viability of cells in a thick collagen gel was improved using a pump-free perfusion system based on differences in water level. In this system, specific devices are not used; instead, a familiar cell culture equipment in a common biological laboratory is employed. This easy setup will help biologists perform perfusion cell culture^24^. Owing to its simplicity, our perfusion system had several limitations. For instance, our system could not regulate the flow rate as perfusion was driven by gravity. Microfluidic devices are introduced when stricter regulations are required.

After perfusion, HepG2 cells maintained their viability in the central part of the collagen gel. Because the flow was generated by gravity in our system, the timing of the medium refill was dependent on the time at which the water-level difference disappeared. In the hand-operated PCC-WLD without syringes, the constant flow was maintained for only 12 h; therefore, we replaced the media to maintain the water level difference every 12 h to improve cell viability. When the medium was refilled every 24 h, cell viability decreased compared to when the medium was refilled every 12 h. Static state in the latter half of a 24 h refill protocol may cause damage to the cells. Flow should be maintained to avoid hypoxia, nutrition shortages, and/or waste retention. In addition, continuous perfusion with the supernatant medium did not improve cell viability. The oxygen concentration, glucose uptake, lactose production, lactate dehydrogenase activity, and/or pH changes in the perfusion medium may also be important for cell survival. In addition, a previous report showed that oxygen concentration decreases with increasing medium depth and affects cellular phenotype^38^. In our 3D perfusion culture system, the gels at the bottom of the medium may have been affected by low oxygen concentrations. However, cell viability, which may be affected by oxygen concentration, was almost 100% in our 3D perfusion culture condition; thus, the oxygen concentration in the gels in our system may be sufficient for cell viability.

During perfusion with fresh medium, cells did not survive after 7 days of culture. In the 7-day culture, the flow rate significantly decreased, and a dense population of cells appeared around the gel surface, which might be due to gel compaction and/or cell proliferation. Liver cells form tight junctions between cells, which act as a barrier to maintain bile in the bile canalicular compartment^39^. HepG2 cells are highly differentiated and reported to form pseudo canaliculi, where small lumens are separated with tight junction proteins between adjacent cells^40^. The decrease in flow rate may be due to the dense population of HepG2 cells, which form a tight junction network. To improve this, blood vessel-like structures must be introduced into the gel, such as hollow fibers^21^ or endothelial tubes. Interestingly, medium perfusion induces endothelial cells to generate perfusable vascular networks^24,41^. In addition, the co-culture of HepG2 cells and endothelial cells has been reported to improve the differentiation of endothelial cells^42^. Therefore, co-culture and perfusion may induce the vascular network around HepG2 cells and help medium perfusion in the dense population of liver cells.

Continuous flow improved cell viability through the depth of the collagen gel, but it was difficult to reestablish the water level difference every 12 h in a hand-operated PCC-WLD system. A syringe was used to create continuous flow for 24 h. The top of the syringe is open in our system, which carries the risk of contamination. In addition, we used aluminum foil, which is easy to tear, as a lid to avoid contamination because we could not use the plastic lid due to the syringe connection. We succeeded in culturing cells in this system for a maximum of 7 days, but this system may be difficult for long-term cultures, such as a month or more. The PCC-WLD system requires long-term manual labor, carries a contamination risk, and has an aluminum lid of low durability, making it less than ideal for long-term culture. Addressing these issues is our next task to allow for a wider range of applications.

The proliferation of and mRNA expression levels of albumin and CYP1A1 in HepG2 cells were higher in a collagen gel than a traditional plastic dish. For the control, we used the cells on plastic culture dishes rather than the cells in collagen gel under static conditions because almost all the cells were dead under static collagen gel conditions. In this experiment, we were not able to distinguish between the effects of 3D culture and the effects of perfusion. Further research is required to evaluate these individually. Furthermore, in our system, the hydrostatic pressure on the cells may be increased by the medium-weight load. A previous report has shown that hydrostatic pressure increases cell proliferation^43^, which may have factored into the results presented here. In addition, HepG2 cells in a 2.4 mg/mL collagen gel had a higher mRNA expression of CYP1A1 than those in a collagen gel with lower concentration. A higher concentration of collagen is well known to increase the stiffness of the gel^36,44^. Stiffness of substrates modulates the expression of various genes and cell behavior^36^. Interestingly, mesenchymal stem cells differentiate into cells of organs with similar stiffness, such as osteoblasts on a hard substrate, myoblasts in the middle, and neurons on a soft substrate^45^. Normal liver tissues have stiffness ranging from 0.91 to 1.55 kPa^44,46^. A plastic dish (2–4 GPa) is markedly stiffer than the liver^47^; therefore, liver genes might be downregulated. Although we did not measure the stiffness of our collagen gels, the 2.4 mg/mL gel may have the closest stiffness to the liver among our gels, which may lead to the upregulation of liver-related gene expression.

Collagen gels have many advantages for cell culture, such as biocompatibility and improvement of cell proliferation and liver function. However, the mechanical weakness of collagen gels, which are easily deformed by external force or cellular contractile force^48^, can be a disadvantage for transplantation. Indeed, the cell-containing collagen gels became compacted after 7-days of culture. To maintain long-term perfusion culture with a collagen gel, mechanical strength of the gel may be required. Genipin is a less cytotoxic crosslinker that increases the mechanical strength of various extracellular matrices, including collagen gels^49–52^. The mechanical reinforcement of collagen gels by genipin treatment may maintain the gel structure during long-term culture.

Our PCC-WLD may be a good exploratory material and screening system for drug cytotoxicity. Two major advantages of our culture system include its ease of use and low cost. Notably, our system does not require complex tube connections or the purchase of various devices. Although the flow rate could not be regulated, the PCC-WLD may serve as a simple perfusion system for evaluating the effect of perfusion in a 3D cell culture.

## Methods

### Cell culture and perfusion cell culture with water level difference (PCC-WLD)

HepG2 cells (RCB1886) were obtained from RIKEN BRC through the National BioResource Project of MEXT/AMED, Japan. HepG2 cells were maintained in Dulbecco’s modified Eagle’s medium (DMEM; Sigma-Aldrich) supplemented with 10% bovine fetal serum (FBS; Sigma-Aldrich). HDFs were obtained from KURABO. HDFs were maintained in 10% FBS and 1% antibiotics (Sigma-Aldrich). The cells were then incubated at 37 °C in a humidified incubator with 5% CO_2_. For the live/dead assay, trypsinized cells were mixed with ice-cold collagen gel solution (Cellmatrix type I-P, Nitta Gelatin Inc.). The cell-gel mixture was placed into 24-well Transwell inserts with 0.4 μm high pore density (CORNING) and then incubated for 30 min at 37 °C for gelation. To determine gel thickness, 200 μL of the gel was measured with a ruler from the side of the insert. For static conditions, 250 μL and 1000 μL of DMEM were added to the inside and outside of the Transwell, respectively. The medium was then replaced daily. The supernatant was prepared by culturing HepG2 cells for 2 days to achieve 50% confluence. For the hand-operated PCC-WLD, the Transwell was set to 24-wells. DMEM (1000 μL) was added to the interior of the Transwell, and the bottom of the Transwell was wet with a small amount of DMEM. For PCC-WLD with a syringe, a 5-mL syringe was connected to the transwell with vacuum grease (DuPont Toray Specialty Materials Kabushiki Kaisha) to avoid leakage of the DMEM. The syringe-connected Transwell was set to a glass ring with a height of 17.5 mm, and the glass ring was placed in a 6-well. Silicon spacers were placed between the glass ring and the bottom of the 6-well to secure the flow path of DMEM. The DMEM trickling through the Transwell to the 6-well was aspirated, and fresh DMEM was added to the syringe every day.

### Analysis of flow rate

In the hand-operated PCC-WLD, the DMEM in the Transwell was collected and weighed at 4, 8, 12 and 24 h after perfusion. After each measurement, the DMEM was returned for subsequent measurements. In the PCC-WLD with a syringe, the volume of the flowing DMEM was measured using the syringe scale. The volume of the flowing DMEM was plotted against the measurement time, and the slope was determined as the flow rate for 24 h. For 7 days, the volume of flowing DMEM was measured; the flow rate was then calculated with the interval of the medium replacement.

### Live/dead cell assay for cells grown in gels in Transwells

To determine whether phalloidin was suitable for the detection of live cells, 2.5 × 10^6^ cells were embedded in 250 μL of 2.4 mg/mL collagen gel. The cell-gel mixture was poured into a glass dish of 1.6 mm diameter and then gelated via 30 min incubation at 37 °C. For the UV-irradiated samples, the cells were exposed to UV light on a clean bench for 1 h and then incubated overnight at 37 °C. For SYTOX Green staining, the cells were stained with 10 μg/mL Hoechst 33342 (Sigma-Aldrich) and 20 nM SYTOX Green (Thermo Fisher Scientific) for 1 h at 37 °C. For phalloidin staining, the cells were fixed with 4% paraformaldehyde in phosphate-buffered saline (PBS) overnight at 4 °C. The gel was permeabilized with 0.5% Triton X-100 in PBS for 15 min and stained with 2 or 10 μg/mL Hoechst and 1:500 diluted Alexa Fluor-488 phalloidin (Thermo Fisher Scientific) in PBS overnight at 4 °C or 3 h at 37 °C. The cells were then observed using a confocal microscope. To observe the cells in the PCC-WLD, 2.0 × 10^6^ cells were embedded in a collagen gel (200 μL of 2.4 mg/mL, if not specified). After fixation with 4% paraformaldehyde in PBS overnight at 4 °C, the gel sample was removed from the Transwell and cut in half in the radial direction using a scalpel to expose the central part of the gel. The gel was placed into 1.5 mL microtubes for staining. For statistical analysis, live cells (phalloidin-positive cells) and dead cells (phalloidin-negative cells) were manually counted using the fluorescent images and ImageJ software.

### Gel shrinkage assay and live/dead cell assay of the cells in floating gels

Using a 24-well plate, 2.0 × 10^5^ cells were embedded in 200 μL of 1.6 mg/mL collagen gel and incubated under static conditions. For floating culture, 4.0 × 10^5^ (HDFs) or 1.0 × 10^6^ (HepG2) cells were embedded in 200 μL of 1.6 mg/ml collagen gel. The cell-gel mixture was poured into a silicone mold (7 mm wide × 7 mm deep × 3 mm high) and then gelated. The gel was removed from the silicone mold and put in wells of a 24-well plate with 1 mL of medium. The 24-well plate was then incubated on a shaker. To evaluate gel shrinkage, the gel area (width × depth) was measured using ImageJ software. To determine cell viability, the gel was stained using the same method as for the live/dead cell assay.

### Proliferation assay

To obtain the calibration curve, 0.1, 0.5, 1.0, 2.0 and 3.0 × 10^6^ cells were seeded on a 6-well plate or into 200 μL of 2.4 mg/mL collagen gel. To generate the calibration curve for the plastic dish culture, the cells were cultured overnight after seeding and then fixed with 4% paraformaldehyde for 10 min. Thereafter, the cells were permeabilized with 0.5% Triton X-100 in PBS for 10 min and stained with 2.5 μg/mL Hoechst 33342 for 1 h at room temperature. The fluorescence intensity of Hoechst was measured using a Fluoroskan FL plate reader (Thermo Fisher Scientific). To generate the calibration curve for the in-gel culture, immediately after completion of gelation, the cell-gel mixture was degraded via incubation with 1 mL of 0.5% collagenase (Wako) in PBS for 1 h at 37 °C. All solutions were then collected and centrifuged at 2000 rpm for 2 min. The supernatant was aspirated, and the cells were stained via resuspension with 2.5 μg/mL Hoechst 33342 in PBS for 1 h at room temperature. The cells were centrifuged, resuspended in PBS, and seeded in a 6-well. Finally, Hoechst fluorescence was detected. When cells proliferate quickly on a plastic dish, overnight incubation after seeding causes underestimation of the cell number due to overestimation in the calibration curve. To evaluate this possibility, 1.0 × 10^6^ cells were seeded and cultured for 24 h and then trypsinized for cell counting. The cell number after the 24-h incubation was 0.97 (± 0.01 S.E.) × 10^6^ cells, which indicated that overnight incubation rarely affected the cell number in the calibration curve. To measure the proliferation rate, 1.5 × 10^5^ cells were cultured on a plastic or in a gel with PCC-WLD for 3 days as described above. Then on plastic and in-gel samples were stained and analyzed using the on-plastic and in-gel calibration curves, respectively. The cell number was calculated using the calibration curves.

### Quantitative real-time PCR

To determine mRNA expression, 1.5 × 10^5^ cells were seeded on cell culture plastic dishes with a diameter of 35 mm or into 200 μL of collagen gels at the indicated concentrations. The plastic dishes were coated with 1:10 diluted collagen (Cellmatrix type I-C, Nitta Gelatin, Inc.) with aqueous HCl (pH = 3.0 HCl aqueous solution) before cell seeding. HepG2 cells were cultured for 3 days on a plastic dish under static conditions or in a collagen gel with PCC-WLD. RNA was extracted using TriPure isolation reagent (Roche). The isolation procedures were performed twice to ensure the removal of any DNA or protein contamination. cDNAs were prepared using the ReverTra Ace kit (TOYOBO), and quantitative PCR was performed using the KAPA SYBR Fast qPCR Kit (NIPPON Genetics Co., Ltd.) and the StepOne Plus real-time PCR system (Applied Biosystems). The following primers were used in this study: S18, 5ʹ-AAGGGTGTGGGCCGAAGATATG-3ʹ (forward) and 5ʹ -GTTCCACCTCATCCTCAGTGAGTTC-3ʹ (reverse); albumin, 5ʹ -TGGTGAAATGGCTGACTGCT-3ʹ (forward) and 5ʹ -CTCTGGTCTCACCAATCGGG-3ʹ (reverse); CYP1A1, 5ʹ -GTCATCTGTGCCATTTGCTTTG -3ʹ (forward) and 5ʹ -CAACCACCTCCCCGAAATTATT-3ʹ (reverse).

### Quantification and statistical analysis

Each experiment was repeated at least 3 times. The error bars represent mean ± S.E. For comparison between two values, both datasets were checked to ensure they follow normal distribution using Kolmogorov–Smirnov test, in which *P* > 0.05 indicates a normal distribution. Thereafter, significantly different variances of two datasets were determined using the *F* test, in which *P* < 0.05 indicates significantly different variance. For datasets without statistically different variances, a two-sided Student’s *t* test was used to analyze the significance. For datasets with statistically different variances, a two-sided Welch’s *t* test was used to determine the statistical significance. For the Student’s *t* test and Welch’s *t* test, *P* < 0.05 indicates statistical significance. For the comparison of data with variance and data without variance, a confidence interval test was used to analyze the significance. For multiple comparisons of the three datasets, the Bonferroni correction was used. For comparison among more than three datasets, each dataset was checked to ensure that it followed a normal distribution using the Kolmogorov–Smirnov test. Statistical significance (*P* < 0.05) was determined using the Tukey–Kramer test.

## Supplementary Information

Supplementary Figures.

## Data Availability

All data generated or analyzed during this study are included in this published article and its supplementary information files.
