# Supplementary material for: Improvement of the cell viability of hepatocytes cultured in three-dimensional collagen gels using pump-free perfusion driven by water level difference
Source: Sci Rep. 2022 Nov 24;12:20269. doi: 10.1038/s41598-022-24423-y (PMC9700666; doi:10.1038/s41598-022-24423-y)
Supplement: Supplementary file 1 — Supplementary Figures. [file 41598_2022_24423_MOESM1_ESM.docx]

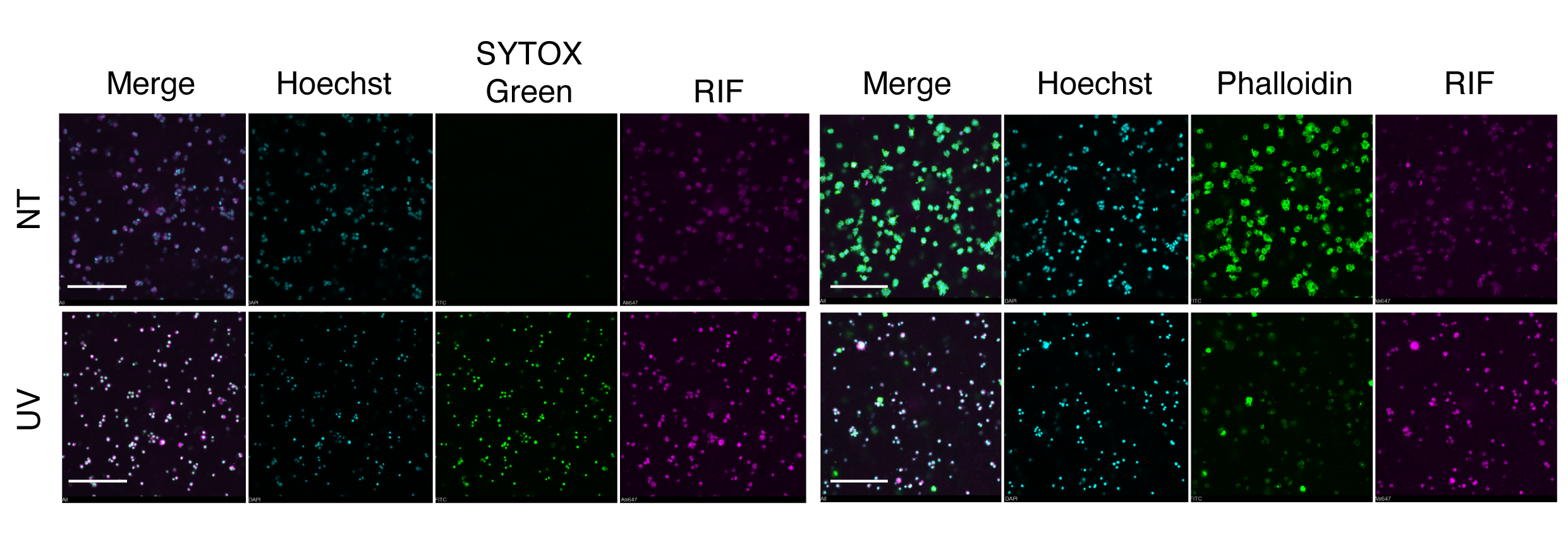


**Figure S1 Phalloidin staining could be used to detect live cells after fixation.**

Fluorescence images of UV-irradiated (UV) and untreated (NT) cells. The cells were not fixed for SYTOX Green staining. For phalloidin staining, the cells were fixed and permeabilized. Cyan, Hoechst. green, SYTOX green, or phalloidin. Magenta, RIF. Scale bar, 200 μm.

**Figure S2 HDFs were viable in thick collagen gel.**

(**a**) Images of the collagen gels with and without HDFs in a 24-well plate. Black and dotted lines indicate the circumference of the well and collagen gel, respectively. Scale bar, 5 mm. (**b**) Images of the cell-containing collagen gels in floating culture. Scale bar, 5 mm. (**c**) The relative area of the gels in the floating culture for HDF and HepG2 cells over time. *n* = 3 independent experiments. Mean ± S.E. (**d**) Fluorescent images of the live/dead cell assay of HDF and HepG2 cells in the collagen gels. Cyan, Hoechst. Green, phalloidin. Magenta, RIF. Scale bar, 200 μm.


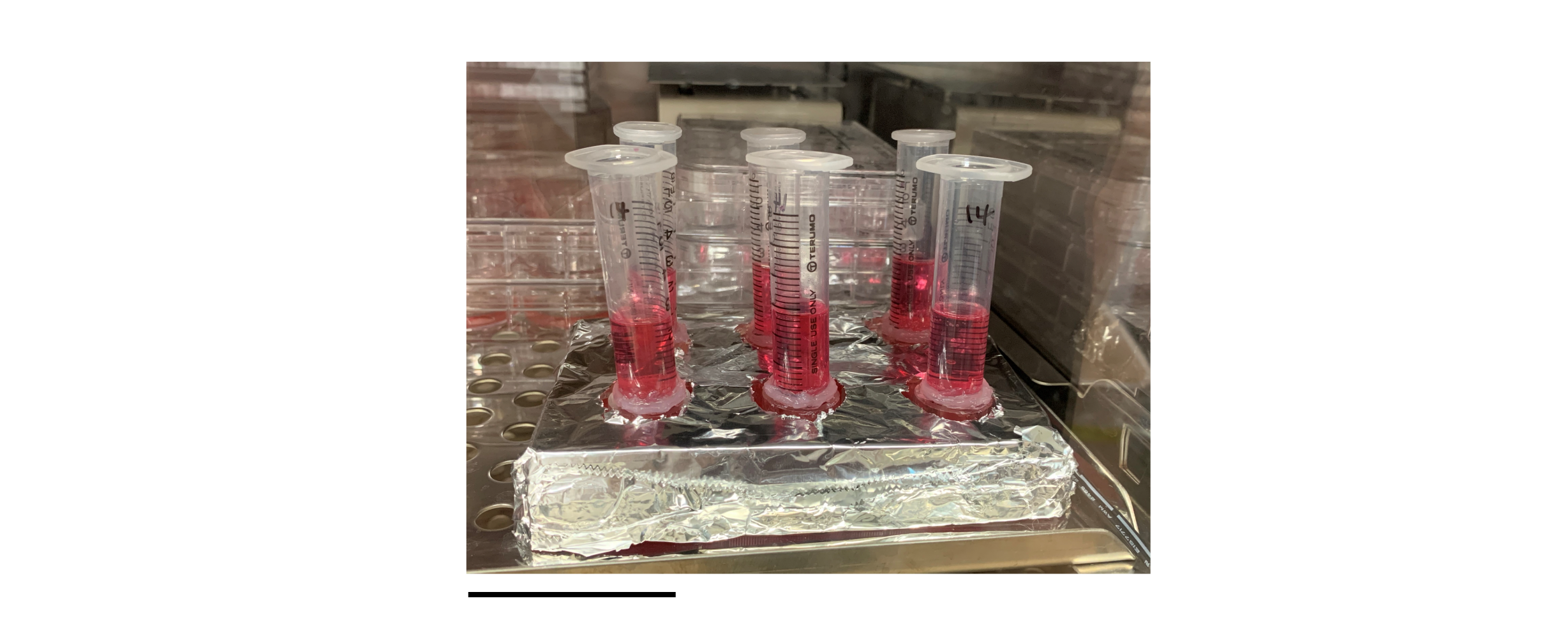


**Figure S3 Image of the PCC-WLD system with syringe.**

Image of the PCC-WLD system using a syringe. The 6-well plate was covered with aluminum foil to prevent bacterial contamination. Scale bar, 5 cm.

**Figure S4 The increase in gel depth and culture period induced cell death in PCC-WLD.**

(**a**) Side view image of the Transwell with different volumes of collagen gel. Samples were cultured for 3 days in PCC-WLD. Scale bar, 5 mm. (**b**) Sectional view of the gel arranged according to the depth from the surface. The percentage of cell viability is depicted on the image above. Scale bar, 200 μm. (**c**) The flow rate of DMEM from the Transwell in PCC-WLD with a syringe for 7 days. *n* = 3 independent experiments. Mean ± S.E. n.s., no significance. *, p < 0.0001 by Tukey-Kramer test against Day 1-2. (**d**) Side view image of Transwell and immunofluorescent image of the cell-gel mixture after 7 days of culture. 200 μL of 2.4 mg/mL collagen gel with/without cells was cultured for 7 days in PCC-WLD. Cyan, Hoechst. Green, phalloidin. Magenta, RIF.
